# A Record Distribution: £230,000

**Published:** 1919-12-20

**Authors:** 


### KING EDWARD'S HOSPITAL FUND FOR LONDON.

### A Record Distribution; £230,000.

A Meeting of the President and General Council of King Edward's Hospital Fund for London for the purpose of awarding grants to the hospitals, convalescent homes, and consumption sanatoria for the present year, was held on Tuesday morning, December 16, at St. James's Palace, H.R.H. The Prince of Wales being in the Chair.

### Total Receipts for Year, £180,000.

**Lord Revelstoke** (the Hon. Treasurer) said that the receipts on general account to December 6, after deducting expenses, amounted to £158,338 11s. 9d. This amount corresponded very closely with the estimate which was before the Council at their meeting on November 7, when the amount to be distributed was determined. The Council would remember that it was anticipated that if the League of Mercy were to be in a position to make the same contribution as in 1918, the total receipts for the year would be approximately £180,000; but that with the help of the undistributed balance of the donation from the late Viscount Astor in 1917 and the receipts from the estate of the late Isabella, Countess of Wilton, in 1916, it would be possible to distribute the sum of £230,000 as recommended by the Executive Committee. This recommendation was adopted at the Council meeting held in November last, and the allocation of this £230,000 was part of the business to which the Council would be invited to give their attention to-day

**Sir William Collins,** in making the annual statement on behalf of the League of Mercy, said he took the opportunity of stating how pleased the League of Mercy were that His Royal Highness had become their Grand President. The amount which the League would be in a position to hand over to the Fund this year was £17,000, being an advance of £1,000 over the figure of last year, and £3,000 over that of the last pre-war year. This would bring the total amount provided by the League to the Fund up to £293,000. In addition, the League had made grants to hospitals within its area of collection outside the County of London amounting to £32,895; making a total of £325,895 paid by the League to the voluntary hospitals in or around London during the last twenty years.

**The Prince of Wales:**

I would wish to thank Sir William Collins for the kind way in which he has referred to my presidency of the League of Mercy. I consider it a very great privilege to become President of the League, and I look forward to presiding at its annual meeting, which will be to-morrow afternoon. I must also thank the League for the splendid sum to be handed over to the King's Fund for the current year.

Sir John Tweedy (the Chairman of the Distribution Committee) presented the Report of that Committee (see p. 263), and Sir Frederick M. Fry (Hon. Secretary) presented the list of awards (see p. 264).

Sir William H. Bennett (the Chairman of the Convalescent Homes Committee) presented the Report of that Committee (see p. 265), and Mr. John G. Griffiths (Hon. Secretary presented the list of awards (see p. 266).

### H.R.H. The President's Speech.

**The Prince of Wales,** in moving the adoption of the Reports and Awards, said :—

My Lords and Gentlemen,

I have first the honour to read the following letter from His Majesty the King :—

Buckingham Palace,
15th December, 1919.

Please convey to the Members of the General Council my hearty congratulations upon the results achieved by the Fund during the past year.

The decision to make specially increased grants this year to the London Hospitals on exceptional grounds consequent upon the War has my cordial approval.

The munificent gift of £250,000 from the Red Cross Society and the Order of St. John comes as a splendid encouragement to all who have at heart the work of the Voluntary Hospitals—while the fact that the Distribution Committee has been asked to undertake the apportionment of this sum is a testimony to public confidence in the Fund.

I trust that we may count upon increased support from the ever-generous public in order to enable such extension of buildings to be carried out as will make it possible for the Hospitals to keep abreast of the times in their great mission of healing and mercy.

In conclusion. I desire to express my gratitude to the Chairmen, Secretaries, and Members of the various Committees for their valuable, untiring and devoted services. George R.I.

### The Original Goal Long Passed.

This is the first distribution meeting since I became President, though I had the pleasure of presiding at the Annual Meeting last May, and I realise more than ever what a big thing the Fund is. When it was founded by King Edward in 1897, it was quite rightly acclaimed as a great success because it distributed £57,000 in its first year. Under my father's presidency the distribution reached £150,000, the sum regarded at the outset as the goal at which to aim. Last year the amount distributed was £200,000. This year it is £230,000. It is no light task to be President of such a Fund; especially as, by the Act of Incorporation, the proceedings of every Council and Committee meeting require the President's personal approval.

### Increased Grants to Meet After-War Conditions.

The first point I wish to make is that the increase in the distribution, from £200,000 to £230,000, is an exceptional increase for a special purpose arising out of the war. In normal times the aim of the Fund has always been to increase the distribution gradually, so that there should be no going back. When the war broke out, however, this rule was departed from, and the distribution was reduced. This was because all new building schemes were suspended, and very little money was wanted for capital grants. Now, at the end of the war, we depart again from the rule and make an exceptionally large increase, because extra money is needed for renewals, repairs, and other building operations which have fallen into arrear. We should not, of course, expect to maintain this exceptional increase in another year unless the Fund is placed by the liberality of the public in a position to do so; but we should be free to start again from the

**King Edward's Hospital Fund—(*continued*).**

more normal basis of 1918, disregarding altogether these fluctuations caused by the war.

CHRISTMAS GIFTS WILL BE WELCOMED.

That the present distribution of £230,000 is exceptional becomes obvious if we compare it with the total estimated receipts, which amount only to £180,000. **It is true that, this time last year, when the Council distributed £6,000 more than its estimated income, the deficit was made up before December 31. If any generous donors feel moved to help us to the same happy result this year, there is still a fortnight left, and I should be most grateful for any Christmas presents to the Fund. In the meantime we have to make these extra grants out of the reserve formed by the legacy of the late Isabella, Countess of Wilton, in 1916, and the balance of the gift of the late Viscount Astor in 1917. But £50,000 is a very deep dip into this reserve, and, if we are to help the hospitals over their difficulties in the next few years without drawing on our permanent funds, we shall need a much larger annual income, which I earnestly hope the charitably disposed will provide.**

The receipts for the year include, besides the annual subscription of £1,000 with which His Majesty the King honours the Fund, contributions of £5,000 from Mr. Walter Morrison, £4,000 from the Drapers' Company, and £1,000 each from the late Viscount Astor, from Sir John Ellerman, from Sir Frederick Green—a member of the Distribution Committee, and from the Merchant Taylors' Company; and the magnificent total of £17,000 contributed by the League of Mercy. I am pleased to see that the share, so kindly allocated to the Fund, of the profits of the Charity Cricket Match, Surrey *versus* Middlesex, played at the Oval last July, came to £385. I saw that match myself. Legacies have included a further £10,000 from the estate of the late Brigade-Surgeon John Law, £5,000 left by the late Mr. James B. Dugdale, and a further £5,000 from the estate of the late Mr. Samuel Lewis, which has now added no less than £472,000 to the capital of the Fund. (Hear, hear.)

THE EXPANSION OF THE VOLUNTARY HOSPITALS.

The Distribution Committee again draw attention to the large number of extension schemes put forward by the hospitals. Last year the Committee announced their intention of making a complete survey of all such schemes, and invited the hospitals to send in particulars. The returns to date show a total of about 1,000 beds already provided since 1913, nearly 3,000 definitely contemplated, and more than 1,000 foreshadowed in the future. The Distribution Committee estimate that these extensions would cost at least £3,000,000 to build, and half a million a year to maintain. It is no wonder that the Committee wish to consider the general effect of all the schemes taken as a whole. I do not want, and I am sure the Distribution Committee do not want, to discourage the prudent extension of voluntary hospitals. We of this Council believe in the voluntary hospital system, which the King's Fund has done, and is doing, so much to maintain. (Hear, hear.) The voluntary hospitals ought to expand as the demand increases for the services they are best fitted to supply. But the financial resources of the voluntary system are not unlimited, and though by energetic cultivation they can be made to grow, the process of expansion must be conducted with prudence, or there may be a real risk of a breakdown. (Hear, hear.)

SPECIAL DISTRIBUTION FROM BRITISH RED CROSS FUNDS.

This prudent expansion of the voluntary hospitals will be greatly assisted by the action of the Joint Committee of the British Red Cross Society and the Order of St. John of Jerusalem, in placing at the disposal of the Fund the large sum of £250,000 out of their surplus funds. Under the conditions laid down by the Joint Committee this is to be applied, on their behalf, to definite schemes for extensions or improvements at hospitals, in the area of the Fund, which can and will treat ex-sailors and ex-soldiers. I am sure that everybody will be keen about that, and will realise what our duty is to those men. The special distribution will thus assist, not only such schemes of extension as pass the scrutiny of the Distribution Committee, but also schemes for the improvement of existing accommodation. The British Red Cross Society and the Order of St. John are doing a great service to the voluntary hospitals by giving them a share of their surplus. By entrusting the King's Fund with the allocation of £250,000, they recognise the value of the special knowledge which it possesses, and, in gratefully accepting their offer, we have gladly placed this knowledge at their disposal. The Fund has never before accepted any gift subject to conditions which would prevent the General Council from distributing it in the same proportions as the general distribution. Sir Ernest Cassel's great gifts of £31,500 and £28,000 were no exception to this rule, since they were given to increase the ordinary grants by a fixed percentage. But we have willingly made an exception in the case of the Red Cross surplus funds; and have agreed to the same conditions as those laid down by the Joint Committee for the rest of the country. I wish to thank them most warmly, in your name and in my own, for their generous action. (Hear, hear.)

SMALLER INSTITUTIONS.

The Convalescent Homes Committee have been allotted a larger sum than last year, and have thus been enabled to meet the claim of the Homes which had, during the war, been either turned into military hospitals or closed because of air-raids. The Committee have also been able to increase the number of beds secured in consumption sanatoria for the use of London hospitals. Unfortunately, they have, so far, found it impossible to get as many beds for women patients as are asked for by the hospitals.

There have been very few changes of personnel during the year, but the Fund has lost the services of an able visitor by the death of Mr. H. B. Irving.

THE PRINCE'S THANKS.

It now only remains for me to express my thanks to the Distribution Committee and the Convalescent Homes Committee, together with the visitors and other officers, for all the work they have done. I would specially thank the Speaker of the House of Commons, Lord Cambridge, and Lord Iveagh, for having exercised on my behalf, during my absence overseas, the powers of President, which, as Governors, they exercised for so many years in their own names. I was fortunate in being able to leave the affairs of the Fund in such experienced hands. I now move the adoption of the reports. (Hear, hear.)

**The Lord Mayor (Sir Edward Cooper)** having seconded the motion, the adoption of the reports was put, and carried unanimously.

On the motion of Sir Alfred Keogh, seconded by the Chairman of the London County Council (Lord Downham), it was resolved "that the cheques for the grants should be posted on Friday, December 19, except in the case of grants for the reservation of beds in consump-

**King Edward's Hospital Fund—*(continued)*.**

tion sanatoria during 1920, which should be posted on January 1.

### ARRANGEMENTS FOR 1920.

**The Prince of Wales then said :**

My Lords and Gentlemen,—I have now to introduce the business relating to 1920. In exercise of my powers as President, I intend to reappoint all the present members of the Council and Committees and officers. The draft resolutions delegating powers to the Committees are before you. If they are approved, the meeting of next year's Council to adopt them need only be formal. The increase in the estimate for office expenses is not wholly caused by the general rise in prices. It is partly due to the fact that there will be two distributions—the ordinary distribution in the autumn, and the distribution of the £250,000 from the Red Cross Society and the Order of St. John. This is to be quite separate, and made at a different time of year. It will mean a great deal of extra work for the Distribution Committee, and most of this will come in 1920, since the Joint Committee wish the grants to be paid over to the hospitals by March 31, 1921. They consider that by that time the schemes should be matured, although of course the actual building need not be begun so soon.

There was moved by the Chief Rabbi (the Rev. J. H. Hertz, D.D.), seconded by the President of the Royal College of Physicians (Sir Norman Moore, Bt.), and approved, a resolution delegating powers to the Finance Committee, Distribution Committee, and Convalescent Homes Committee.

On the motion of Viscount Knutsford, seconded by the President of the Royal College of Surgeons (Major-General Sir George H. Makins), a resolution was approved delegating further powers to the Finance Committee.

On the motion of Viscount Farquhar, seconded by Sir Walter Lawrence, Bt., a resolution was approved delegating powers to the Executive Committee.

Viscount Sandhurst moved a vote of thanks to His Royal Highness for presiding.

Sir Horace Marshall, in seconding the resolution, said how delighted the Council felt that His Royal Highness had returned to this ountry in such excellent health, and how much they thanked him for the great work he had done for the British Empire.

The resolution was carried by acclamation.

### H.R.H.'s Plans.

**The Prince of Wales, in acknowledging the vote of thanks, said : "I am certainly very glad indeed to be back in the old country. I had a wonderful time in Canada, but, as probably most of you know, I am not going to be here very long now. His Majesty the King is sending me to Australia and New Zealand, which will necessitate my leaving this country probably about March and being away six or seven months. That will mean that I shall miss the meeting of this Council in May. As to my movements later in the year, I have no idea at the present moment, but I look forward to meeting the Council again, I hope, on my return from my next journey." (Hear, hear.)**

The proceedings then terminated.

